# Link Between Anhedonia, Reward Dysfunction, and Eating Behavior Across Mental and Physical Health Conditions: A Narrative Review

**DOI:** 10.3390/nu18121981

**Published:** 2026-06-18

**Authors:** Beata Ewa Grønbæk, Derek V. Byrne, Barbara Vad Andersen

**Affiliations:** 1 Food Quality Perception and Society Team, iSense Lab, Department of Food Science, Aarhus University, Agro Food Park 48, DK 8200 Aarhus, Denmark; beena@food.au.dk (B.E.G.); derekv.byrne@food.au.dk (D.V.B.); 2 Sino-Danish College (SDC), University of Chinese Academy of Sciences, Beijing 101408, China

**Keywords:** anhedonia, pleasure, individual differences, eating behavior

## Abstract

**Background/Objectives**: Anhedonia, the inability to derive pleasure from typically enjoyable activities, is prevalent in psychiatric and neurological disorders, and is linked to conditions such as obesity and eating disorders. Simultaneously, characteristics of altered eating behavior have been observed amongst these segments, yet the link between anhedonia and eating behavior, and its mechanisms remain underexplored. **Methods**: This current narrative review synthesizes evidence for a relationship between pleasure perception and eating behavior among individuals with anhedonic traits, emphasizing the role of reward processing and its variations across different segments. **Results**: Our narrative review identified specific segments with anhedonic traits and described the link to characteristics of their eating behavior. **Conclusions**: This research can significantly contribute to a better understanding of the differences in the relationship between pleasure and eating behavior amongst individuals with anhedonic traits, which is crucial for developing effective treatment strategies for individuals affected by anhedonia and disturbed eating.

## 1. Introduction

Anhedonia, commonly defined as the inability to experience pleasure or enjoyment from normally pleasurable activities, remains prevalent in the general population over shorter and longer time periods as a symptom of various mental and neurological disorders. It is especially common in conditions such as major depressive disorder, schizophrenia, Parkinson’s disease, substance use disorder, Alzheimer’s disease, and eating disorders [1,2,3,4]. Despite its centrality in these disorders, anhedonia is not consistently recognized as a primary symptom across diagnostic manuals. For instance, the World Health Organization’s International Classification of Diseases (ICD-10) includes “loss of interest and pleasurable feelings” as a non-essential symptom of depressive episodes without explicitly naming anhedonia [4].

Anhedonia also has broader implications beyond just the inability to experience pleasure—it disrupts reward-related functions, for instance, motivation to engage in pleasurable activities and the ability to make decisions based on past rewarding experiences [4]. This broader understanding of anhedonia, shaped by advances in cognitive psychology and behavioral neuroscience, highlights its role in more complex deficits in reward processing, encompassing difficulties with anticipation, consumption, and learning from rewards. These impairments are particularly pronounced in individuals with major depressive disorder and schizophrenia, where anhedonia extends beyond the absence of pleasure to more intricate disruptions in reward processing [2,3].

It is further important to note that anhedonic traits are not limited to clinically diagnosed individuals. They are also present amongst the general population, including those experiencing stress or depression-like symptoms. These traits are present in a population that, from a clinical perspective, is considered “normal”, though the prevalence of anhedonia in this group remains poorly understood [5]. Recent research has shown that individuals suffering from chronic stress exhibit signs of anhedonia-driven changes in eating behavior, namely changes in appetite, altered perceptions of food pleasure, and shifts in eating behavior [6]. A study found that 65% of participants experiencing moderate stress had normal hedonic scores, whereas only 31% of participants with high stress reported the same, indicating a higher prevalence of anhedonia in the high-stress group (*p* < 0.001). Further, it has been found that acute psychosocial stress can increase cravings for highly palatable foods for some, while for others an experience of loss of appetite prevails [7]. Overall, these studies suggest that milder or even temporary stress can lead to anhedonia-like eating behavioral symptoms [6]. As such, it can be hypothesized that anhedonic traits extend beyond clinical conditions, affecting populations that may be considered “normal” but still experience disruptions in reward processing, particularly around food-related behaviors.

Food-related anhedonia, or the reduced pleasure from eating, has gained increased attention, particularly in relation to conditions like obesity and eating disorders. While there is evidence that food anhedonia may be linked to disruptions in the brain’s reward system, particularly dopaminergic pathways, our understanding of its mechanisms and long-term impact on eating behavior and psychological well-being remains incomplete [3,8]. Disruptions in reward processing are thought to contribute to individuals seeking out primary reinforcers, such as for example food, as a compensatory mechanism for deficiencies in the reward system [8,9].

Studies show that the impaired ability to perceive pleasure varies significantly among different segments of the population with anhedonic traits. Therefore, in this narrative review, we aim to explore the prevalence of anhedonia amongst different population segments. Moreover, food-related behaviors have been found to differ among these segments, hence a secondary aim of the narrative review is to clarify the specific food choice characteristics and eating patterns associated with these segments.

Given the rising global prevalence of mental health disorders, many of which are comorbid with obesity, there is an urgent need to deepen our understanding of the complex relationship between anhedonia, mental health, and eating behaviors [10,11,12]. This research can significantly contribute to understanding and developing more effective treatment strategies for individuals suffering from both psychiatric and eating disorders, which are often intertwined with reward-processing deficits.

## 2. Theories of Anhedonia

### 2.1. Capacity Theory

The prevailing psychological theory of anhedonia, which continues to have substantial support, is relatively straightforward: individuals with anhedonia suffer from a reduced hedonic capacity [13,14]. Hedonic capacity refers to the maximum level of positive affect a person can experience in daily life [15]. According to this capacity theory, anhedonia is characterized by significantly lowering this upper limit of pleasure, resulting in anhedonic individuals’ inability to experience the same level of pleasure as non-anhedonic individuals, leading to a diminished sense of enjoyment from previously pleasurable activities [15]. A study conducted by Fiorito and Simons (1994) supports this theory, as they found that anhedonic individuals report positive images as less positive and experience positive and negative images as less emotionally impactful than those without anhedonia [16]. Additionally, physiological indicators of emotional arousal, such as changes in heart rate, and behavioral responses, such as facial expressions, are reduced in anhedonic individuals [16]. These findings suggest that anhedonia may be associated with a diminished capacity to experience and express emotional responses to rewarding stimuli.

### 2.2. Sustainability Theory

Although the capacity theory remains influential, several studies indicate that the core issue in anhedonia may not lie entirely in hedonic capacity, or at least not exclusively [17]. The sustainability theory points out that individuals with anhedonia are capable of experiencing the same peaks of enjoyment as those not suffering from the condition. However, their ability to sustain these pleasurable responses over time is significantly impaired [17,18]. The central argument is that while pleasurable reactions to stimuli are generally temporally extended, in cases of anhedonia, the duration of positive affect is notably reduced. A study conducted by Heller and colleagues (2009) found that brain regions involved in reward processing and regulating positive affect exhibited a marked decrease in activation in response to positive stimuli over time in individuals with anhedonia. In contrast, control subjects maintained consistent activation levels [17]. These findings suggest that anhedonia may involve deficits in sustaining positive emotional states rather than an inability to generate them. Having said that, current research highlights that anhedonia and its multifaceted nature are recognized as a core feature of reward deficits in various segments with anhedonic traits, particularly among individuals with schizophrenia, MDD, and diabetes [4,19].

### 2.3. Implications for Eating Behavior and Reward Processing

These theoretical perspectives provide a useful framework for understanding how anhedonia may influence eating behavior and food-related reward processing. According to the capacity theory, reduced hedonic capacity may diminish the rewarding value of food, potentially contributing to decreased enjoyment of eating and restrictive eating behaviors [15]. Sustainability theory suggests that individuals may experience normal initial pleasure from food consumption but have difficulty maintaining positive affect over time, potentially leading to compensatory reward-seeking behaviors [17,18].

However, it remains unclear whether eating-related disturbances associated with anhedonia are primarily driven by impairments in anticipatory reward (“wanting”), consummatory reward (“linking”), reward learning, or a combination of these processes. Therefore, understanding the conscious and subconscious reward processes involved in eating may help clarify how anhedonia contributes to altered eating behavior and food-related decision-making.

### 2.4. Conscious and Subconscious Reward Mechanisms in Eating Behavior and Anhedonia

To gain a deeper understanding of reward in relation to eating behavior, specifically, it is essential to examine the conscious and subconscious reward processes involved in eating, as well as potential disruptions within these processes. Conscious reward processing refers to the subjective awareness and cognitive evaluation of rewarding experiences, including the anticipation of food rewards and the conscious experience of pleasure during food consumption. In contrast, subconscious reward processing involves automatic motivational, emotional, and physiological responses to rewarding stimuli that occur without conscious awareness and may influence food preferences, cravings, and learned associations with food. Both conscious and subconscious reward processes contribute to eating behavior and may be altered in individuals with anhedonia [2].

The concept of “hedonia,” derived from the Greek word “Hedone,” meaning pleasure, refers to the ability to experience pleasure [3]. In contrast, the French psychologist Ribot introduced the term “anhedonie” to describe the inability to experience pleasure [20]. To accurately conceptualize and measure food-related pleasure, it is essential to differentiate between the terms “reward” and “pleasure” and their subcomponents, namely “wanting,” “liking,” and “learning.” A “reward” is a natural process through which the brain associates various stimuli with a positive or desirable outcome. Meanwhile, “pleasure” is conceptualized as a positive psychological response that may be experienced consciously or subconsciously and is essential in maintaining a normal sense of well-being [2]. The reward system in humans is activated by various pleasurable stimuli involving brain circuitry, particularly the metabolic system/dopaminergic pathway, which is essential for humans to physiologically and cognitively process the reward. Dopamine release increases the pleasurable experience of the reward and assigns a positive or negative value to it [2]. Since reward experiences are rather subjective, the timing, context, and circumstances surrounding rewarding experiences can significantly affect behaviors related to the reward process [21,22,23].

Various problematic eating behaviors are hypothesized to originate from an imbalance within the reward system. Reward and anhedonia can be conceptualized as parts of a cyclical process comprising appetitive (also referred to as “anticipatory”), consummatory (also referred to as “experienced”), and satiation (also referred to as “learning”) phases [24]. In the context of food intake, the term “wanting” is often used, referring to the subconscious and conscious desire to obtain a food-related reward experienced predominantly in the pre-consumption “appetitive/anticipatory” phase. The term “liking” comprises both the subconscious and conscious experience of pleasure derived from obtaining the rewarding stimulus, thus relating to the consummatory phase. Finally, the “learning” subconsciously links the experiences of wanting (anticipatory pleasure) and liking (consummatory pleasure) over time, allowing for future predictions of the anticipation values of a food experience. Hormones involved in the homeostatic system, such as leptin, ghrelin, and insulin, show significant influence on the mesolimbic dopaminergic system, which plays a key role in regulating “wanting,” “liking,” and “learning” and, thereby, the overall pleasure derived from food [2]. From this perspective, anhedonia may result from a deficiency in processing any of these subcomponents [25,26]. Disturbances in these processes may contribute to deficits in pleasure and overall reward sensitivity, expressed in different eating patterns [27,28]. Individual differences in reward sensitivity, either reduced or heightened, have been suggested as a possible underlying factor in particular eating disorders, stress-induced eating, overeating, and obesity [29].

### 2.5. Drivers of Pleasure in Food-Related Contexts and Individual Differences

Andersen et al. (2021) proposed a concept for measuring the pleasurable aspects of eating and how pleasure is involved in eating behavior and food choices. In the article, the authors further clarified how drivers of pleasure and perceived food pleasure can vary among individuals yet tend to form characteristic patterns across populations [30]. Research indicates that various factors, including the food itself, perceptions, and sensations linked to its consumption, values, personal lifestyle, and health-related circumstances, affect pleasurable aspects of eating and, thus, shape unique and subjective profiles, referred to as “food pleasure profiles.” These profiles reflect how individuals assign varying degrees of importance to different elements that drive pleasure in food-related contexts [8]. As lifestyle and health conditions affect the ability to experience pleasure, and these conditions are subject to potential treatment, it is possible that individual food pleasure profiles are dynamic, too, and can vary across contexts and over time. Yet, this is still to be researched.

### 2.6. Segments with Anhedonic Traits and Pronounced Eating Behavioral Characteristics

The inability to perceive pleasure varies significantly across individuals, both those with and without diagnoses, and is closely linked to overall health, particularly mental health [4]. Mental health conditions, such as mood disorders, depression, schizophrenia, and acute stress, have been associated with alterations in eating behavior, underscoring the importance of understanding how psychological factors and anhedonic traits influence the perception of food-related pleasure [4,31]. Numerous studies reported that people suffering from mental illnesses are more likely to experience being unpleasantly aroused and unmotivated for life’s experiences (negative affect), resulting in positive, rewarding experiences being purposely avoided and possibly resulting in depressive symptoms in the future [25]. For instance, patients suffering from depression and schizophrenia have been associated with alterations in anticipatory- and consummatory rewards, often leading to problematic eating behaviors, such as binge eating, restrictive eating, and an inability to find food pleasurable driven by higher levels of wanting and lower (or unaltered) levels of liking [3,8]. Similarly, anhedonia, a common symptom of Major Depressive Disorder, is associated with a decline in health-related quality of life and psychosocial functioning, largely due to impairments in the reward system [32,33]. The dysfunction in the reward system has been proven to cause metabolic disturbances, with studies suggesting that obesity significantly moderates the association between MDD and willingness to experience reward-related behaviors, particularly as a consequence of increased anticipatory reward responses [34]. In addition, anxiety disorders are likewise a group of mental disorders that have been associated with anhedonic traits, although not with the same level of severity as in MDD. Although anxiety is often considered a symptom of MDD, it may also lead individuals to intentionally avoid pleasurable experiences, resulting in the development of depressive symptoms over time [25].

Eating disorders, in particular, have been consistently linked to both general and social anhedonia, although the expression of anhedonic traits and reward sensitivity varies depending on the type of disorder [35,36]. Additionally, higher levels of anhedonia have been found to predict long-term weight gain [37]. One proposed explanation is that diminished reward experience may promote compensatory food-seeking behavior aimed at increasing reward exposure. Consistent with this hypothesis, individuals with high BMI often show higher wanting (i.e., anticipatory reward), despite relatively normal liking (i.e., consummatory reward), which leads to a constantly increased search for food [3,8]. Similarly, people with binge-eating disorder experience abnormally high levels of wanting when exposed to food, even when they do not particularly enjoy it [27,28]. Heightened reward sensitivity can result in the overconsumption of highly palatable foods in individuals with bulimia nervosa or binge-eating disorder, mainly due to increased motivation for the activities that offer immediate rewards, whereas reduced sensitivity to food pleasure may contribute to food avoidance in those with anorexia nervosa [9,28].

Besides the well-documented link to depression, schizophrenia, and other mood disorders, anhedonia is also common among patients with Alzheimer’s disease, Parkinson’s disease, and substance use disorders [4]. Therefore, depending on the nature of the disease, varying degrees of anhedonic traits are prominent in many people’s life quality and health status. Consequently, understanding how anhedonic traits affect eating behavior is crucial for addressing public health challenges, particularly obesity, type 2 diabetes, and mental illness [3].

## 3. Materials and Methods

Peer-reviewed literature published between 2000 and 2024 was included in this narrative review, with a focus on the prevalence of anhedonia and its relationship with eating behavior among different segments. Studies conducted as either a systematic review, a meta-analysis approach, or a clinical study design were considered for inclusion, whereas book chapters and brief introductory articles about anhedonia were excluded. Literature was sourced from databases, including Google Scholar and PubMed.

The search was conducted using the following keywords: “anhedonia,” “anhedonic traits,” “eating behavior,” “food choices,” “diabetes,” “major depressive disorder,” “obesity,” “segments with anhedonic traits,” “mental illnesses,” and “schizophrenia.” Boolean operators (“AND”, “OR”) were used to combine search terms. Examples of search strings included: (“anhedonia” OR “anhedonic traits”) AND (“eating behavior” OR “food choices”), (“anhedonia”) AND (“obesity” OR “prediabetes” OR “diabetes”), and (“anhedonia”) AND (“schizophrenia” OR “major depressive disorder” OR “bipolar disorder” OR “eating disorders” OR “Parkinson’s disease”). Searches were restricted to English-language, peer-reviewed publications published between [2000] and [2024]. To ensure relevance and quality research, the inclusion criteria were as follows: (1) anhedonia among various segments, (2) obese segments with prediabetes (chosen as a specific interest topic as prediabetes and obesity are starting to become a focus in anhedonia research). Exclusion criteria included papers that lacked the aspect of anhedonia.

**Figure 1:**
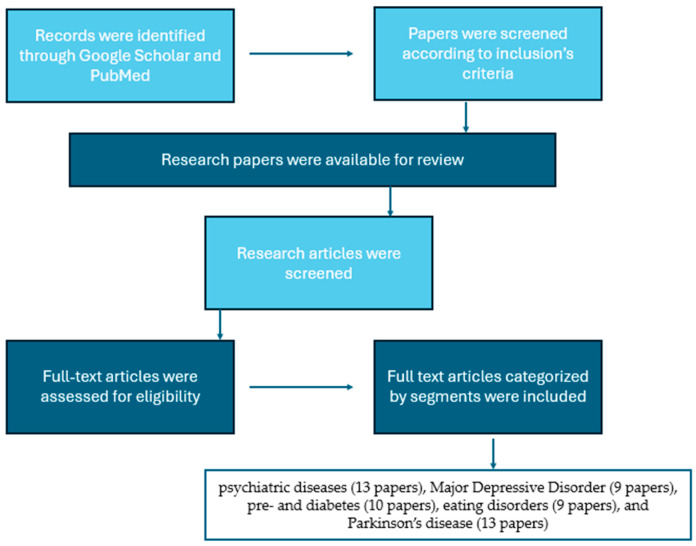
Example of the literature search.

Moreover, for each selected paper, the key points, such as segment type, methodology, and main findings regarding anhedonia and eating behaviors, were extracted from each study. Thematic coding was used to categorize articles by segments and main findings related to anhedonia and eating behavior, respectively (e.g., psychiatric diseases (13 papers), Major Depressive Disorder (nine papers), prediabetes and diabetes (10 papers), eating disorders (nine papers), and Parkinson’s disease (13 papers)). Furthermore, qualitative thematic and content analyses were conducted to identify similarities between segments and their relations to anhedonia. Figure 1 shows a detailed example of the literature search.

This section will present the results of the literature study regarding the prevalence of anhedonia among various segments with anhedonic traits and whether these segments show specific eating behavioral characteristics. The following segments are in focus: individuals with psychiatric illnesses (schizophrenia and bipolar), Major Depressive Disorder (depression and anxiety), prediabetes and diabetes, eating disorders, and Parkinson’s disease.

### 3.1. Psychiatric Illnesses (Schizophrenia and Bipolar)

#### 3.1.1. The Prevalence of Anhedonia Among Psychiatric Illnesses (Schizophrenia and Bipolar)

The literature search resulted in four studies that investigated the relationship between the prevalence of anhedonia and psychiatric illnesses. These findings are shown in Table 1.

Anhedonia, the diminished ability to experience pleasure, is a core symptom of several psychiatric illnesses, particularly schizophrenia and bipolar disorder, and it is a prominent symptom of their clinical picture [38]. Schizophrenia is a severe and chronic psychiatric disorder characterized by disruptions in thought processes, perceptions, and emotional regulation, and frequently presents with negative symptoms, including anhedonia. Studies indicate that anhedonia is present in approximately 40–60% of patients with schizophrenia, with some reports suggesting even higher prevalence in specific clinical populations [39]. In the study by Pelizza and Ferrari (2009), anhedonia was found in 45% of all patients, yet in the depressed sample, anhedonia was reported by 36.9% of subjects [38]. To a lesser degree, anhedonia is also prevalent in bipolar disorder, particularly during depressive episodes [38]. In a study involving 58 bipolar patients, significant correlations were found between anhedonia, biological rhythm disturbances, and depression severity [40]. Additionally, approximately 20–25% of patients with mood disorders report anhedonic symptoms. Research about the neurobiological mechanisms underlying anhedonia in schizophrenia and bipolar illnesses suggests that the prevalence of anhedonia is caused by dysregulation in reward processing systems, which can vary depending on the patient’s mood state [41].

**Table 1:** Prevalence of anhedonia and psychiatric illnesses.

Authors/Title	Aim	Methodology/Participants	Main Findings Regarding Anhedonia
Pelizza & Ferrari, 2009 [38]. “Anhedonia in schizophrenia and major depression: state or trait?”	The aim was to examine the pathological features of anhedonia in schizophrenic and depressed patients, and to investigate its clinical relations with general psychopathology (negative, positive, and depressive dimensions).	Clinical study/145 patients (80 schizophrenics and 65 depressed subjects)	In the schizophrenic sample, anhedonia reached high significant levels only in 45% of patients (*n* = 36). This ‘anhedonic’ subgroup was distinguished by high scores in the disorganization and negative dimensions. Positive correlations of anhedonia with disorganized and negative symptoms have also been detected. In the depressed sample, anhedonia reached high significant levels in only 36.9% of subjects (*n* = 24). This ‘anhedonic’ subgroup is distinguished by high scores in the depression severity and negative dimensions.
Horan, Kring, & Blanchard, 2006 [39]. “Anhedonia in schizophrenia: a review of assessment strategies”.	The aim was to describe the 3 major approaches that have been used to assess anhedonia in schizophrenia.	Meta-analysis	It is concluded that anhedonia can be reliably assessed and constitutes a distinctive, clinically important aspect of schizophrenia that should be included in a comprehensive evaluation of negative symptoms.
Gorostowicz, Chrobak, Dudek, & Siwek, 2024 [40]. “Relationship between anhedonia, biological rhythms, functioning and depression severity in patients with bipolar disorder”.	The aim was to study interactions between anhedonia, dysregulation of biological rhythms and functioning/depression severity in patients with bipolar disorder are demonstrated for the first time.	Clinical study/58 patients	The study found statistically significant correlations between anhedonia, disruptions in biological rhythms, functioning, and the severity of depression in patients with bipolar disorder. This indicates that these factors are interrelated and can influence each other. Both biological rhythm dysregulation and anhedonia were identified as independent predictors of the level of functioning and the severity of depression.
Rizvi, Lambert & Kennedy, 2018 [41]. “Presentation and Neurobiology of Anhedonia in Mood Disorders: Commonalities and Distinctions”.	The aim was to focus on the clinical and behavioral presentation of anhedonia in mood disorders, as well as the differences and commonalities in the underlying neurocircuitry.	Clinical study/291 patients	In a clinical sample of 291 patients, unipolar depressed patients exhibited greater severity of anhedonia compared to those with bipolar disorder. Approximately 20–25% of patients with mood disorders report residual anhedonic symptoms even after treatment, indicating that anhedonia may persist as a trait factor in these disorders. The prevalence of anhedonia can also be observed in youth at risk for mood disorders.

#### 3.1.2. Eating Behavior Among Segments with Psychiatric Illnesses (Schizophrenia and Bipolar)

In total, nine studies in the literature were found to have investigated the relationship in the eating behavior among patients with psychiatric illnesses, as shown in Table 2.

Individuals diagnosed with psychiatric illnesses, particularly schizophrenia and bipolar disorder, often exhibit distinct food-related behaviors and dietary patterns.

Eating behaviors among patients with schizophrenia are complex and often disordered, influenced by various biopsychosocial factors. In the study by Sankaranarayanan et al. (2021), patients with schizophrenia frequently present disordered eating behaviors (DEBs), particularly binge eating (4.4% to 45%), food cravings (16.1% to 64%), food addiction (27% to 60.6%), and night eating (4% to 30%). These problematic eating behaviors can significantly contribute to nutritional deficiencies and/or excessive weight gain, significantly impacting their health and quality of life [42]. Moreover, a study by Khosravi (2020) further found that 41.5% of schizophrenia patients exhibited DEBs, compared to 10.3% in a control group [43]. Research indicates that individuals with schizophrenia are more likely to exhibit unhealthy dietary patterns, characterized by high consumption of processed foods, sugars, and fats and a lower intake of fruits, vegetables, and fiber [44]. These problematic dietary preferences are often intensified by negative symptoms of the disorder, such as apathy and lack of motivation, which can diminish the individual’s capacity to prepare healthy meals or make conscious food choices [45]. Moreover, a common symptom of schizophrenia, suspiciousness, is associated with food refusal, inappropriate feeding behaviors, and increased prevalence of DEBs [46]. In the study by Osuji & Onu (2019), food refusal was seen in 56.5% of the patients, with 32.5% attributed to suspiciousness. In contrast, among the 13.2% with inappropriate feeding behavior, 46.4% were related to abnormal food preparation [46]. While suspiciousness and paranoid ideation may contribute to food refusal in some patients, negative symptoms such as anhedonia and reduced motivation may also influence eating behavior. Impairments in reward processing can reduce the anticipated pleasure associated with food consumption, potentially contributing to diminished interest in eating, irregular eating patterns, and reduced motivation to engage in food-related activities [42,43,45,46]. Furthermore, the study by Mueller-Stierlin et al. found that patients with schizophrenia commonly experience disrupted hunger cues and emotional eating rather than physical hunger, leading to binge eating, night eating, food cravings, and emotional eating [47]. Here, Khosravi (2020) points out that, in general, emotional factors such as stress, anxiety, and depression are known to influence eating behaviors, especially among schizophrenia patients, which can further exacerbate DEBs [43]. Lastly, the use of atypical antipsychotic medications is well documented to affect weight gain and increase appetite, with higher rates of binge eating and food cravings, especially for calorie-dense foods, among patients with schizophrenia [42,43].

Eating behaviors among patients with bipolar disorder vary significantly during manic and depressive episodes, reflecting the complex interplay between mood states and eating patterns. During manic episodes, individuals may exhibit impulsive eating behaviors, increased appetite, and a preference for high-calorie foods [48,49]. A study by Koning et al. (2022) found that patients with manic episodes often experience increased appetite and cravings for high-energy foods. Moreover, there is also a tendency for impulsive eating, which can lead to binge-like behaviors or erratic eating schedules [48]. Studies suggest that BD patients show a preference for unhealthy, high-sugar, and high-fat foods [49] and are generally seeking out high-reward foods (often high in sugar or fat) as a way to improve their mood temporarily [48]. In contrast, depressive episodes often followed by emotional eating led to reduced appetite, irregular eating patterns, and potential weight gain. Many patients engage in emotional eating, with food being used as a coping mechanism to deal with negative emotions. This can significantly contribute to changes in weight, including weight gain, which are frequently observed among these patients due to altered eating habits [50]. However, many patients reported a significant decrease in appetite, leading to skipped meals. A study by Kilbourne et al. (2007) reported that patients with bipolar disorder report poor eating behaviors, including eating only one meal per day and difficulty obtaining or cooking food, which particularly increased during depressive episodes [49].

**Table 2:** Eating behavior among segments with psychiatric illnesses.

Authors/Title	Aim	Methodology/Participants	Main Findings Regarding Eating Behavior
Sankaranarayanan, Johnson, Sanop, Mammen, Wilding, Vasani, Murali, Mitchison, Castle, & Hay, 2021 [42]. “Disordered Eating among People with Schizophrenia Spectrum Disorders: A Systematic Review”.	The aim was to systematically review literature on disordered eating among people with schizophrenia spectrum disorder (SSD).	Meta-analysis/31 studies	The reported rates were 4.4% to 45% for binge eating, 16.1% to 64%, for food craving, 27% to 60.6% for food addiction, and 4% to 30% for night eating. Positive associations were re-ported for binge eating with antipsychotic use and female gender, between food craving and weight gain, between food addiction and increased dietary intake, and between disordered eating and female gender, mood and psychotic symptoms. Reported rates for disordered eating among people with SSD are higher than those in the general population.
Khosravi, 2020 [43]. “Biopsychosocial factors associated with disordered eating behaviors in schizophrenia”.	The aim was to investigate biopsychosocial factors associated with DEBs in schizophrenia.	Cross-sectional study/308 participants	The prevalence of DEBs was 41.5% in schizophrenic patients (vs. 10.3% in the control group, *p* = 0.012). No significant difference was observed in the EAT-26 scores based on gender and phases of schizophrenia. According to multiple linear regression analysis, lack of psychosocial rehabilitation, use of atypical antipsychotics, early stages of psychosis, high level of anxiety and depression, expression of more active psychotic symptoms, tobacco smoking, and suffering from type 2 diabetes were all associated with increased development of DEBs among schizophrenic patients.
Teasdale, Ward, Samaras, Firth, Stubbs, Tripodi, & Burrows, 2019 [44]. “Dietary intake of people with severe mental illness: Systematic review and meta-analysis”.	The aim was to conduct a systematic, comprehensive evaluation of the published research on dietary intake in psychotic disorders and bipolar disorder.	Systematic meta-analysis/58	People with SMI were found to have significantly higher dietary energy (mean difference 1332 kJ, 95% CI 487–2178 kJ/day, *p* = 0.002, g = 0.463) and sodium (mean difference 322 mg, 95% CI 174–490 mg, *p* < 0.001, g = 0.414) intake compared with controls. Qualitative synthesis suggested that higher energy and sodium intakes were associated with poorer diet quality and eating patterns.
Strassnig, Brar, & Ganguli, 2003 [45]. “Nutritional assessment of patients with schizophrenia: a preliminary study”.	Impact of anhedonia in schizophrenia.	Clinical study/146 patients	Schizophrenia patients as a group ate more food when compared to NHANES III subjects, but the relative percentages of calories derived from fat, protein, and carbohydrates were not found to be different.
Osuji & Onu, 2019 [46]. “Feeding behaviors among incident cases of schizophrenia in a psychiatric hospital: Association with dimensions of psychopathology and social support”.	The aim was to describe the various eating behaviors among incident cases of schizophrenia and its relationship with the dimensions of psychopathology and perceived social support.	Cross-sectional study/206 participants	Food refusal was seen in 56.5% of the patients, with 32.5% of it attributed to suspiciousness. Of the 13.2% with inappropriate feeding behavior, 46.4% and 14.3% were related to abnormal food preparation and pica, respectively. Food refusal was significantly associated with positive symptoms dimension and general psychopathology.
Mueller-Stierlin, Peisser, Cornet, Jaeckle, Lehle, Moerkl, & Teasdale, 2022 [47]. “Exploration of Perceived Determinants of Disordered Eating Behaviors in People with Mental Illness—A Qualitative Study”.	The aim was to understand the perceived determinants of eating behaviors, in particular those connected to disordered eating patterns, in people with SMI.	Cross-sectional study/28 semi-structured interviews	Participants reported a lack of daily structure, time, and drive, which hindered their ability to maintain healthy eating habits. Many participants experienced difficulties in recognizing their feelings of hunger and satiety. Emotional factors played a significant role in eating behaviors. Participants reported using food as a coping mechanism, driven by motives such as reward, social influences, and conformity. This emotional eating led to behaviors like binge eating, night eating, and cravings for specific foods.
Kilbourne, Dana, Rofey, McCarthy, Post, Welsh, & Blow 2007 [49]. “Nutrition and exercise behavior among patients with bipolar disorder”.	The aim was to investigate the nutrition and exercise habits of patients diagnosed with bipolar disorder (BPD) compared to those with schizophrenia and individuals without serious mental illness (non-SMI).	Cross-sectional design/6710 patients	Patients with BPD were more likely to report poor exercise habits, including infrequent walking (odds ratio, OR ¼ 1.33, *p* < 0.001) or strength exercises (OR ¼ 1.28, *p* < 0.001) than those with no SMI. They were also more likely to self-report suboptimal eating behaviors, including having fewer than two daily meals (OR ¼ 1.32, *p* < 0.001) and having difficulty obtaining or cooking food (OR ¼ 1.48, *p* < 0.001). Patients with BPD were also more likely to report having gained +10 pounds in the past 6 months (OR ¼ 1.59, *p* < 0.001) and were the least likely to report that their health care provider discussed their eating habits (OR ¼ 0.84, *p* < 0.05) or physical activity (OR ¼ 0.81, *p* < 0.01). were the least likely to report that their health care provider discussed their eating habits (OR ¼ 0.84, *p* < 0.05) or physical activity (OR ¼ 0.81, *p* < 0.01).
Hirte et al., 2022 [50]. “Eating Habits and Eating Disorder Associated Behavior in Bipolar Disorder”.	The aim was to investigate the relationship between bipolar disorder (BD) and eating disorders (EDs).	Cross-sectional design/86 patients with BD and 86 healthy controls	Higher rates of all EDSSs were reported in BD. Younger individuals with BD showed higher expression in “bulimic symptoms,” “body image and slimness ideal,” and “atypical binge” subscales. No participants fulfilled an ED diagnosis. The findings show a link between EDSS and BD.
Koning, Vorstman, McIntyre & Brietzke, 2022 [48]. “Characterizing eating behavioral phenotypes in mood disorders: a narrative review”	The aim was to synthesize evidence for eating behavioral phenotypes in mood disorders.	Systematic review	The review highlights the complexity of eating behaviors in mood disorders and advocates for a more detailed exploration of these phenotypes to improve clinical outcomes and contribute to the subtyping of mood disorders.

### 3.2. Major Depressive Disorder

#### 3.2.1. The Prevalence of Anhedonia Among Segments with Major Depressive Disorder and Its Co-Occurring Symptoms of Depression and Anxiety

In total, five studies in the literature were found to have investigated the relationship between the prevalence of anhedonia among individuals with Major Depressive Disorder and its co-occurring symptoms, such as depression and anxiety. Table 3 summarizes these studies.

The prevalence of anhedonia within Major Depressive Disorder is notably high, with studies estimating that it affects between 37% and 80% of individuals diagnosed with MDD, depending on diagnostic criteria, populations, and assessment methods [38,40]. In the broader context, depression and anxiety disorders are frequently co-occurring symptoms and predictors of relapse in patients with MDD. Research suggests that anhedonia occurs even after other depressive symptoms have remitted [51]. Among individuals with MDD, the presence of anhedonia has been linked to lower quality of life, including reduced motivation and social engagement, which further complicates recovery from depressive episodes [52]. The neurobiological underpinnings of anhedonia involve disruptions in reward processing circuits, particularly in the dopaminergic pathways, which are implicated in both mood regulation and motivation [14].

**Table 3:** Prevalence of anhedonia among segments with Major Depressive Disorder and its co-occurring symptoms of depression and anxiety.

Authors/Title	Aim	Methodology/Participants	Main Findings Regarding Anhedonia
Pelizza & Ferrari, 2009 [38]. “ Anhedonia in schizophrenia and major depression: State or trait?”.	The aims of this study were to examine the pathological features of anhedonia in schizophrenic and depressed patients, and to investigate its clinical relations with general psychopathology (negative, positive, and depressive dimensions).	Clinical study/145 patients (80 schizophrenics and 65 depressed subjects)	In the schizophrenic sample, anhedonia reached high significant levels only in 45% of patients (*n* = 36). This ‘anhedonic’ subgroup was distinguished by high scores in the disorganization and negative dimensions. Positive correlations of anhedonia with disorganized and negative symptoms were also detected. In the depressed sample, anhedonia reached high significant levels in only 36.9% of subjects (*n* = 24).
Gorostowicz, Chrobak, Dudek, & Siwek, 2024 [40]. “Relationship between anhedonia, biological rhythms, functioning and depression severity in patients with bipolar disorder”.	The aim was to study interactions between anhedonia, dysregulation of biological rhythms and functioning/depression severity in patients with bipolar disorder are demonstrated for the first time.	Clinical study/58 patients	The study found statistically significant correlations between anhedonia, disruptions in biological rhythms, functioning, and the severity of depression in patients with bipolar disorder. This indicates that these factors are interrelated and can influence each other. Both biological rhythm dysregulation and anhedonia were identified as independent predictors of the level of functioning and the severity of depression.
Pizzagalli, Iosifescu, Hallett, Ratner, & Fava, 2008 [14]. “Reduced hedonic capacity in major depressive disorder: Evidence from a probabilistic reward task”.	The aim was to investigate reduced hedonic capacity, or anhedonia (the diminished ability to experience pleasure), in individuals with Major Depressive Disorder (MDD).	Clinical study/23 unmedicated subjects who met the DSM-IV criteria for MDD and 25 matched control subjects recruited from the community	Anhedonia is not only prevalent in MDD but is also considered a risk factor that increases vulnerability to developing depression. Individuals with MDD who report higher levels of anhedonic symptoms tend to exhibit a diminished hedonic capacity, which is linked to their overall depression severity. Moreover, the findings indicate that individuals with MDD show specific impairments in reward responsiveness, particularly when anhedonic symptoms are prominent.
Treadway & Zald, 2011 [53]. “Reconsidering anhedonia in depression: Lessons from translational neuroscience”.	The aim is to refine the understanding of anhedonia, identify its neurobiological bases, and improve treatment approaches for MDD by advocating for more precise definitions and translational research methods.	Empirical analyses/1523 subjects	Anhedonia is identified as one of the two required symptoms for diagnosing MDD, alongside depressed mood. The paper notes that approximately 37% of individuals diagnosed with MDD experience clinically significant anhedonia.
Spijker, De Graaf, Bijl, Beekman, Ormel, & Nolen, 2004 [54]. “Functional disability and depression in the general population. Results from the Netherlands Mental Health Survey and Incidence Study (NEMESIS)”.	The aim was to provide a population-level understanding of how depression contributes to functional limitations, which can inform public health strategies, clinical practices, and policies aimed at reducing the burden of depression and improving functional outcomes.	Prospective epidemiological survey/7076 adults	Functional disabilities and absence days in depressed individuals were not found to be associated with duration of depression. Functioning in daily activities improved with longer duration of recovery but social functioning did not.

#### 3.2.2. Eating Behavior Among Segments with Major Depressive Disorder and Its Co-Occurring Symptoms of Depression and Anxiety

The literature search resulted in four studies investigating the relationship between the eating behavior among segments with MDD, as shown in Table 4.

Eating behaviors among patients with Major Depressive Disorder are characterized by significant alterations of disordered eating behaviors, such as binge eating and loss-of-control overeating, with individuals reporting episodes of binge eating between 19% and 44% [51]. Various factors, including neuroendocrine changes, dietary habits, emotional eating, and disordered eating behaviors, significantly affect the prevalence of these problematic eating behaviors and can lead to either weight gain or loss [48,52]. Furthermore, the disruptions in the reward system affect food intake by increasing impulsivity and a higher intake of unhealthy options like lard and sugar [48,55]. A study by Stefańska et al. (2014) found that patients with recurrent depressive disorders consume significantly less healthy foods and essential nutrients, such as fruits, vegetables, and fish, which may exacerbate depressive symptoms [55].

**Table 4:** Eating behavior among segments with Major Depressive Disorder and its co-occurring symptoms of depression and anxiety.

Authors/Title	Aim	Methodology/Participants	Main Findings Regarding Eating Behavior
Hombali, Valli, Chang, Lin, Ong, E., Abdin, Siow, Chong, & Subramaniam 2019 [51]. “Prevalence of disordered eating behaviors in mental illness-a systematic review”.	The aim was to review the empirical studies that have determined the prevalence of disordered eating behaviors in patients with psychiatric illness.	Meta-analysis/13 studies	The evidence suggests that the individuals seeking treatment for various mental illnesses endorsed having disordered eating behaviors such as binge eating, fasting, purging (use of diuretics, diet pills and laxatives; vomiting and excessive exercise), loss of control overeating, bulimic tendency and anorexia. The prevalence ranges of binge eating (19.0–44.0%), bulimic tendency (4.4–13.2%), anorexia (19.8%), fasting (4.0–40%), laxative use (1.0–11.1%), diuretic use (1.0–1.6%), vomiting (1.6–8.6%), diet pills (36.0%), loss of control overeating (17.0%) and excessive exercise (1.0–32.0%) were reported in the included studies.
Mills, Larkin, Deng, & Thomas 2021 [52]. “Cortisol in relation to problematic eating behaviors, adiposity and symptom profiles in Major Depressive Disorder”.	The aim was to explore the complex relationships between cortisol levels, eating behaviors, body weight, and symptoms of Major Depressive Disorder (MDD).	Clinical study/37 participants with MDD weight loss, 43 participants with MDD appetite/weight gain, and 60 healthy subjects	The results indicate that cortisol is related to lower indices of adiposity and depressogenic symptoms of appetite/weight loss but is not related to problematic eating behaviors and appetite increases in MDD. These findings provide further evidence that the melancholic and atypical subtypes of MDD are associated with differential neuroendocrine and anthropometric indices, as well as behavioral and symptom profiles.
Stefańska, Wendołowicz, Cwalina, Kowzan, Konarzewska, Szulc, & Ostrowska, 2014 [55]. “Assessment of dietary habits of patients with recurrent depressive disorders”.	The aim of this study was the evaluation of selected dietary habits of patients with recurrent depressive disorders. methods.	Clinical study/150 patients (75 patients suffering from recurrent depressive disorders and 75 healthy people)	It has been shown that in the compared groups of women, patients with depression consumed significantly less groats (*p* < 0.001), rice (*p* = 0.02), red meat (*p* < 0.01), fish (*p* < 0.01), vegetables (*p* < 0.001), fruits (*p* < 0.01) and wine (*p* < 0.001) in comparison with women without depression, and they were significantly more likely to consume wheat-rye bread (*p* = 0.03), cheese (*p* = 0.02), butter (*p* = 0.03), cream (*p* < 0.01), lard (*p* < 0.001), coffee (*p* = 0.03) and sugar (*p* = 0.02) in comparison with women without depression. Statistically significant differences between the two groups of men were diagnosed in the frequent intake of lard (*p* < 0.001) and less frequent intake of vegetable oils (*p* < 0.01), beer (*p* = 0.01), and fast food (*p* < 0.01) for men with depression compared with men in the control group.
Koning, Vorstman, McIntyre, & Brietzke, 2022 [48]. “Characterizing eating behavioral phenotypes in mood disorders: a narrative review”	The aim was to synthesize evidence for eating behavioral phenotypes in mood disorders.	Systematic review	The review highlights the complexity of eating behaviors in mood disorders and advocates for a more detailed exploration of these phenotypes to improve clinical outcomes and contribute to the subtyping of mood disorders.

### 3.3. Prediabetes and Diabetes

#### 3.3.1. The Prevalence of Anhedonia Among Prediabetic and Diabetic Individuals

The literature search resulted in six studies investigating the relationship between the prevalence of anhedonia among prediabetic and diabetic individuals. These findings are shown in Table 5.

The prevalence of anhedonia is significantly higher in individuals with diabetes, particularly type 2 diabetes, than in the general population, with various studies reporting prevalence rates up to 30% [56,57]. The prevalence appears even higher in individuals with comorbid depressive symptoms, which are also more common among those with diabetes [58]. For example, a study by Shevchuk et al. (2019) on 190 males found that depressive disorders, including anhedonia, were diagnosed in 10.7% of patients with type 2 diabetes, with 12.1% experiencing moderate to severe depressive symptoms [59]. Moreover, a study explored the biological mechanisms, primarily dysregulation of the hypothalamic–pituitary–adrenal (HPA) axis, chronic inflammation, and insulin resistance, which are common in diabetes and major depressive disorder and further affect dopaminergic pathways related to the brain’s reward system, thus contributing to anhedonia [60,61]. Although research highlights that the prevalence of anhedonia in prediabetes remains limited, studies have demonstrated that individuals with prediabetes exhibit elevated levels of depressive symptoms and an increased likelihood of developing mood disorders [58].

**Table 5:** Prevalence of anhedonia among prediabetic and diabetic individuals.

Authors/Title	Aim	Methodology/Participants	Main Findings Regarding Anhedonia
Anderson, Freedland, Clouse, & Lustman, 2001 [56]. “The prevalence of comorbid depression in adults with diabetes: a meta-analysis”	To estimate the odds and prevalence of clinically relevant depression in adults with type 1 or type 2 diabetes.	Meta-analysis	The prevalence of comorbid depression was significantly higher in diabetic women (28%) than in diabetic men (18%), in uncontrolled (30%) than in controlled studies (21%), in clinical (32%) than in community (20%) samples, and when assessed by self-report questionnaires (31%) than by standardized diagnostic interviews (11%).
Hermanns, Schmitt, Gahr, Herder, Nowotny, Roden, Ohmann, Kruse, Haak, Kulze, 2015 [57]. “The effect of a Diabetes-Specific Cognitive Behavioral Treatment Program (DIAMOS) for patients with diabetes and subclinical depression: results of a randomized controlled trial”.	The aim is to reduce diabetes distress.	Cognitive behavioral intervention/214 subjects	The 12-month follow-up revealed a significantly stronger reduction in depressive symptoms (Center for Epidemiologic Studies Depression Scale score) in the DIAMOS group compared with the CG (Δ3.9 [95% CI 0.6–7.3], *p* = 0.021).
Golden, Lazo, Carnethon, Bertoni, Schreiner, Diez Roux, Lyketsos, C., & Greenland, 2008 [58]. “Examining a bidirectional association between depressive symptoms and diabetes”.	To examine the bidirectional association between depressive symptoms and type 2 diabetes.	Multi-Ethnic Study of Atherosclerosis, a longitudinal, ethnically diverse cohort study/4847 subjects	In analysis 1, the incidence rate of type 2 diabetes was 22.0 and 16.6 per 1000 person-years for those with and without elevated depressive symptoms, respectively. The risk of incident type 2 diabetes was 1.10 times higher for each 5-unit increment in CES-D score (95% confidence interval [CI], 1.02–1.19) after adjustment for demographic factors and body mass index.
Shevchuk, Tsiganenko, Taranenko, Kryzhevsky, & Mankovsky, 2019 [59]. “Frequency of Depressive States in Patients with Type 2 Diabetes Mellitus”.	The aim was to investigate the frequency of depressive states in patients with type 2 diabetes mellitus (DM).	Clinical study/190 subjects	The study found that depressive disorders were diagnosed in 10.7% of the patients with type 2 DM. Among the patients diagnosed with depressive disorders, 12.10% exhibited moderate to severe depressive symptoms, as assessed by the PHQ-9 scale.
Raison, Capuron, & Miller, 2006 [60]. “Cytokines sing the blues: Inflammation and the pathogenesis of depression”.	The study aims to explore the role of cytokines—proteins involved in immune signaling and inflammation—in the development (pathogenesis) of depression.	Meta-analysis	Increasing amounts of data suggest that inflammatory responses have an important role in the pathophysiology of depression. Depressed patients have been found to have higher levels of proinflammatory cytokines, acute-phase proteins, chemokines and cellular adhesion molecules.
Egede & Ellis, 2010 [61]. “Diabetes and depression: Global perspectives”.	The aim was to investigate diabetes and depression.	Systematic review of the literature	Diabetes and depression are debilitating conditions that are associated with significant morbidity, mortality, and healthcare costs. Coexisting depression in people with diabetes is associated with decreased adherence to treatment, poor metabolic control, higher complication rates, decreased quality of life, increased healthcare use and cost, increased disability and lost productivity, and increased risk of death.

#### 3.3.2. Eating Behavior Among Prediabetic and Diabetic Individuals

In total, four studies in the literature were found to have investigated the eating behavior in prediabetic and diabetic segments, as shown in Table 6.

Eating behaviors among people suffering from diabetes, particularly those with type 2 Diabetes (T2D), significantly influence dietary intake and their overall health. Research indicates that this population shows signs of either emotional, external, or restrained eating patterns, respectively affecting their dietary choices and nutritional outcomes. Emotional eaters tend to consume more calories and fats, with women mainly showing a higher intake of non-alcoholic beverages such as soft drinks and juices and men a higher intake of alcohol [62,63]. Additionally, emotional and disordered eating were linked to antidepressant use, indicating varied eating behaviors among diabetic individuals, particularly females [64]. Furthermore, patients who eat in response to external cues also exhibit increased consumption of calorie-dense foods and fat, with women showing higher emotional and restrained eating tendencies than men [63]. Lastly, among individuals suffering from diabetes, both male and female, restrained eating is characterized by conscious control over food intake. Research showed mixed results; it is not clearly correlated with dietary intake but is associated with longer diabetes duration and higher body mass index [63,64]. Further, binge eating and night eating commonly occur among diabetic patients, leading to excessive food intake and complicating glycemic control and weight normalization [65].

**Table 6:** Eating behavior among prediabetic and diabetic individuals.

Authors/Title	Aim	Methodology/Participants	Main Findings Regarding Anhedonia
Arhire, Gal, Gherasim, Nita, Popa, Mihalache, & Graur, 2024 [62]. “Associations between Eating Behavior and Dietary Intake in a Sample of Type 2 Diabetes Patients”.	The aim of this study was to evaluate how eating behavior affects dietary intake in a population of patients with T2DM using validated tools.	Cross-sectional quantitative study/416 subjects	Emotional eaters and external eaters showed a significantly higher intake of calories, lipids, nonalcoholic beverages, such as soft drinks and juices (in women) and alcohol (in men). There were no correlations between restrained eating and dietary intake.
Gal, Iatcu, Popa, Arhire, Mihalache, Gherasim, Nita, Soimaru, Gheorghita, Graur, & Covasa, 2024 [63]. “Understanding the Interplay of Dietary Intake and Eating Behavior in Type 2 Diabetes”.	The aim was to explore the relationship between dietary intake and eating behavior among patients with type 2 diabetes mellitus (T2DM).	Cross-sectional study/416 subjects	Women scored significantly higher than men for emotional and restrained eating (*p* < 0.001). Correlation analyses showed that emotional eaters consumed significantly more calories (r = 0.120, *p* = 0.014) and fat (r = 0.101, *p* = 0.039), as well as non-alcoholic beverages for women (r = 0.193, *p* = 0.003) and alcohol for men (r = 0.154, *p* = 0.038). Also, individuals who ate based on external cues consumed significantly more calories (r = 0.188, *p* < 0.001) and fat (r = 0.139, *p* = 0.005).
Eymael, Borges, Pandolfo, Farias, Martins Filho, Kilpp, Leal, Castro, Torres, & Bertacco, 2022 [64]. “Eating behavior in outpatients with type 2 diabetes mellitus and/or systemic arterial hypertension: a cross-sectional study”.	The aim is to evaluate the eating behavior of patients diagnosed with diabetes and/or hypertension who are receiving care in a nutrition outpatient clinic.	Cross-sectional retrospective design/55 subjects	Among the different domains of eating behavior assessed, cognitive restraint was found to have the highest median score. The study identified a significant association between emotional eating and gender, with female patients showing a higher tendency towards emotional eating (*p* = 0.0079). Uncontrolled eating behavior was significantly associated with the use of antidepressant medications (*p* = 0.0403).
Laniush & Urbanovych, 2020 [65]. “Eating disorders in patients with type 2 diabetes”.	This review analyzes the impact of eating disorders on type 2 diabetes mellitus.	Meta-analysis	The review identifies binge eating and night-eating syndrome as the most common eating disorders among patients with type 2 diabetes. A notable finding is that many patients with binge eating and night-eating syndrome are often unaware of their eating problems.

### 3.4. Eating Disorders

#### 3.4.1. The Prevalence of Anhedonia Among Segments with Eating Disorders

In total, six studies in the literature were found to have investigated the relationship between the prevalence of anhedonia among patients with eating disorders. Table 7 summarizes these studies.

Anhedonia, a common symptom of mood disorders, has been increasingly recognized by various studies as an essential symptom of eating disorders [53]. Recent research highlights the high prevalence of anhedonia across various forms of eating disorders, including anorexia nervosa, bulimia nervosa, and binge-eating disorder. Anhedonia in these populations is often related to disturbances in reward processing, leading to problematic eating behaviors, such as restrictive eating and binge eating [66]. Studies consistently show elevated rates of anhedonia among individuals with EDs compared to the general population. For instance, Harrison et al. (2010) found that individuals with anorexia nervosa display significant reductions in the ability to experience pleasure from both food-related and non-food-related activities. In their sample, over 50% of individuals with AN reported moderate to severe anhedonia [67]. Similarly, Levinson et al. (2017) observed high levels of anhedonia in individuals with bulimia nervosa, particularly during periods of restrictive dieting or post-binge guilt [68]. In a study examining patients with eating disorders, Dolan et al. (2021) reported that individuals with EDs reported the prevalence of anhedonia to be notably higher compared to healthy individuals and further found that it may be linked to the severity of the disorder [69]. These findings suggest that anhedonia is not only prevalent but may play a pivotal role in the maintenance of problematic eating behaviors. Neurobiological studies have suggested that alterations in dopamine pathways, critical for reward processing, may underlie the co-occurrence of anhedonia and eating disorders, particularly in AN and BN [70].

**Table 7:** Prevalence of anhedonia among segments with eating disorders.

Authors/Title	Aim	Methodology/Participants	Main Findings Regarding Anhedonia
Kaye, Fudge, & Paulus, 2009 [66]. “New insights into symptoms and neurocircuit function of anorexia nervosa”	The aim is to explore and clarify the underlying neurobiological mechanisms associated with anorexia nervosa (AN).	Literature review	New insights into anorexia nervosa (AN) reveal that symptoms may stem from primary disturbances in brain appetitive circuits rather than solely psychosocial influences. Brain imaging studies indicate altered serotonin and dopamine metabolism, with specific dysfunctions in the insula affecting interoceptive processing and striatal activity influencing reward modulation.
Treadway & Zald, 2011 [53]. “Reconsidering anhedonia in depression: Lessons from translational neuroscience”.	The primary aim of the paper is to enhance the understanding of anhedonia, particularly in the context of major depressive disorder (MDD), by refining its definition and exploring its neurobiological underpinnings.	Meta-analysis	Anhedonia is recognized as a core symptom of MDD, alongside depressed mood. The paper notes that approximately 37% of individuals diagnosed with MDD experience clinically significant anhedonia. It is mentioned that MDD has a lifetime prevalence of about 16%, indicating that a substantial portion of the population may experience this disorder at some point in their lives.
Levinson et al., 2017 [68]. “The core symptoms of bulimia nervosa, anxiety, and depression: A network analysis”.	The study had two primary aims: (a) to identify which symptoms of BN are central to the disorder and (b) to test which symptoms of anxiety and depression are most strongly related to symptoms of BN.	Randomized controlled trial	Results showed that fear of weight gain was central to BN psychopathology, whereas binge eating, purging, and restriction were less central in the symptom network. Symptoms related to sensitivity to physical sensations (e.g., changes in appetite, feeling dizzy, or wobbly) were identified as bridge symptoms between BN and anxiety and depressive symptoms.
Harrison, Sullivan, Tchanturia, & Treasure, 2010 [67]. “Emotional functioning in eating disorders: attentional bias, emotion recognition and emotion regulation”.	The study aims to investigate how individuals with eating disorders process emotions.	Clinical study/190 women—50 with AN, 50 with BN and 90 healthy controls (HCs)	Those with an ED showed attentional biases to faces in general (medium effect), but specifically to angry faces over neutral faces (large effect) compared to HCs. The ED group also reported significantly higher emotion regulation difficulties (large effect) than HCs. There was a small difference between the ED and HC groups for the emotion recognition task (small-medium effect), particularly in the restricting AN (RAN) group.
Dolan, Khindri, Franko, Thomas, Reilly, & Eddy, 2021 [69]. “Anhedonia in eating disorders: A meta-analysis and systematic review”.	The study aims to provide both a quantitative and qualitative synthesis of the existing literature on this topic.	Systematic review	Findings indicated that anhedonia is elevated in EDs and may be a relevant treatment target.
Kaye, Wierenga, Bailer, Simmons, & Bischoff-Grethe, 2013 [70]. “Nothing tastes as good as skinny feels: the neurobiology of anorexia nervosa”.	The study aims to explore the underlying neurobiological mechanisms associated with anorexia nervosa.	Meta-analysis	Neurobiological studies have suggested that alterations in dopamine pathways, critical for reward processing, may underlie the co-occurrence of anhedonia and eating disorders, particularly in AN and BN.

#### 3.4.2. Eating Behavior Among Segments with Eating Disorders

The literature search resulted in three studies investigating the relationship between eating behavior among segments with eating disorders, as shown in Table 8.

Eating behaviors among individuals with eating disorders vary significantly across different segments, reflecting a spectrum of disordered eating patterns. These behaviors can be categorized into typical and atypical eating patterns, such as anorexia nervosa, bulimia nervosa, and binge eating disorder. Research indicates that AN patients engage in severe caloric restrictions or extreme dieting practices [71]. On the other hand, bulimia nervosa participants reported fewer meals and frequent overeating, while binge-eating disorder participants had higher snack frequency [72]. Yet, both groups reported atypical eating behaviors like nibbling and consuming double-portion meals. Moreover, BED patients showed a higher frequency of binge eating episodes, with more frequent breakfast consumption linked to lower BMI [72]. Having said that, the study by Samarina et al. (2015) suggests that across individuals with eating disorders, there is a tendency towards irregular meal patterns and increased snacking, which can increase the prevalence of disordered eating behaviors [73].

**Table 8:** Eating behavior among segments with eating disorders.

Authors/Title	Aim	Methodology/Participants	Main Findings Regarding Anhedonia
Kontic, Vasiljevic, Jorga, Jašović-Gašić, Lakić, & Arsic, 2010 [71]. “Presence of different forms of compensatory behaviors among eating disordered patients”.	The aim was to determine the presence of various inappropriate compensatory behaviors among patients with eating disorders, specifically comparing them to a control group of healthy individuals.	Clinical study/35 female eating-disordered patients and 70 females in control group	A high statistically significant difference existed in the presence of all compensatory behaviors in the experimental and control group, regarding vomiting (χ^2^ = 40.6; *p* < 0.001), misuse of laxatives and diuretics (χ^2^ = 33.7; *p* < 0.001), extreme dieting (χ^2^ = 23.4; *p* < 0.001) and excessive exercising (χ^2^ = 27.1; *p* < 0.001).
Masheb, Grilo & White, 2011 [72]. “An examination of eating patterns in community women with bulimia nervosa and binge eating disorder”.	To better understand the eating patterns of persons with eating disorders.	Clinical study/bulimia nervosa (BN; *n* = 39), binge eating disorder (BED; *n* = 69), or controls (CON; *n* = 203).	In terms of typical eating behaviors, the BN group ate significantly fewer meals, particularly lunches, than the other two groups. Atypical eating, such as nibbling, eating double meals and nocturnal eating, was significantly more common in the eating disorder groups. More frequent breakfast consumption was associated with lower BMI in the BED and CON groups, and more frequent meal consumption was associated with less binge eating in the BED group only.
Samarina, Sharp & La, 2015 [73]. “Eating Disorders and Eating Disordered Behaviors”.	The aim was to explore the complexities surrounding eating disorders and the behaviors associated with them.	Meta-analysis	The paper discusses a range of disordered eating behaviors, which include dieting, extreme weight control methods (such as fasting, binge eating, and purging), and clinically diagnosed eating disorders.

### 3.5. Parkinson’s Disease

#### 3.5.1. Prevalence of Anhedonia Among Segments with Parkinson’s Disease

In total, five studies in the literature were found to have investigated the relationship between the prevalence of anhedonia among patients with Parkinson’s disease. Table 9 summarizes these studies.

Various scales and methods have been used to study the prevalence of anhedonia among patients with Parkinson’s disease. Overall, it has been reported that among PD patients, the prevalence rate of anhedonia varies between 16.5 and 70%, depending on the assessment procedure and the study population [74]. A study by Reichmann et al. (2003) on 657 subjects investigated depressive symptoms in Parkinson’s disease and the prevalence of anhedonia among PD patients. The study explicitly found that 46% of PD patients were anhedonic. Moreover, they found a significant positive correlation between anhedonia, depression, and the severity of PD symptoms. The relation between anhedonia and depression in PD patients is found among the majority of the studies from the literature search. It seems to indicate that anhedonia in this segment is at least partly mediated via depression [75]. As an example, research conducted by Lemke et al. discovered a higher prevalence of anhedonia in depressed PD patients than in non-depressed PD patients, with 79.7% of depressed PD patients and 36.1% of non-depressed PD patients classified as anhedonic [76], and the study conducted by Weintraub et al. found that PD patients did not demonstrate anhedonia when they experienced positive life events [77]. Fibiger studied anhedonia as a symptom of depression in patients with PD. He found that the functions of specific reward-related systems are impaired among depressed PD patients. Specifically, the author emphasizes that the reduced ability to experience the pleasure of reward is a key symptom of clinical depression among PD patients [78].

**Table 9:** Prevalence of anhedonia among segments with Parkinson’s disease.

Authors/Title	Aim	Methodology/Participants	Main Findings Regarding Anhedonia
Grover, Somaiya, Kumar & Avasthi, 2015 [74]. “Psychiatric aspects of Parkinson’s disease”.	The aim of this review is to look at the existing information on various psychiatric conditions, like depression, psychosis, anxiety disorders, sleep disturbances and cognitive deficits in patients with PD.	Systematic review, meta-analysis approach	Existing data suggests that PD should not be considered only as a motor disorder. In addition to the motor symptoms, patients with PD have a very high prevalence of non-motor symptoms. Among the various non-motor symptoms, psychiatric manifestations are very common and are associated with impairment in quality of life and higher treatment costs.
Reichmann, Brecht, Koster, Kraus & Lemke, 2003 [75]. “Pramipexole in routine clinical practice: a prospective observational trial in Parkinson’s disease”.	The aim of this study was to confirm the beneficial effects of pramipexole on the core symptoms of Parkinson’s disease (with a focus on tremor), as well as to assess its antidepressant activity, during routine clinical practice.	Prospective observational study/657 subjects	The study explicitly found that 46% of PD patients were anhedonic. Moreover, they found a significant positive correlation between anhedonia, depression, and the severity of PD symptoms. The relation between anhedonia and depression in PD patients is found among the majority of the studies from the literature search. It seems to indicate that anhedonia in this segment is at least partly mediated via depression.
Lemke, Brecht, Koester, Kraus & Reichmann, 2005 [76]. “Anhedonia, depression, and motor functioning in Parkinson’s disease during treatment with pramipexole”.	The aim was to assess the frequency of anhedonia in patients with idiopathic Parkinson’s disease and the relationship of anhedonia and parkinsonian motor deficits during treatment with pramipexole.	Clinical study/657 subjects	Mild depression was present in 47% of the patients and moderate to severe depression in 22%. Anhedonic individuals included 45.7% of all patients and 79.7% of depressed Parkinson’s disease patients. Anhedonic Parkinson’s disease patients had greater motor deficits, restrictions in activities of daily living, and depression compared to non-anhedonic patients.
Weintraub, Cary, Stern, Taraborelli & Katz, 2006 [77]. “Daily affect in Parkinson disease is responsive to life events and motor symptoms”.	The aims of this study were to examine the daily affective experiences of patients with Parkinson disease (PD) and to determine their association with daily events and motor symptoms.	Clinical study/24 non-depressed male subjects and 23 healthy male subjects	Overall, patients with PD reported significantly less positive and more negative affect than healthy peers over time. There were similar, and expected, associations between negative events and affect in both groups. Although patients with PD reported far fewer positive events than control subjects, they reported as great an improvement in affect in response to them.
Fibiger, 1984 [78]. “The neurobiological substrates of depression in Parkinson’s disease: a hypothesis”.	The aim is to explore and propose potential neurobiological mechanisms underlying depression in individuals with Parkinson’s disease (PD).	Literature Review	Since Parkinson’s disease involves dopamine depletion due to degeneration of dopaminergic neurons in the substantia nigra, the study may hypothesize that dopamine deficiency contributes not only to motor symptoms but also to mood disorders like depression. Dopamine pathways, especially those that connect to limbic regions, might play a role in mood regulation. Fibiger might suggest that beyond dopamine, other neurotransmitters such as serotonin and noradrenaline—which are also implicated in mood disorders—may be dysregulated in PD. This would offer a broader neurochemical basis for depression in PD.

#### 3.5.2. Eating Behavior Among Segments with Parkinson’s Disease

In total, eight studies in the literature were found to have investigated eating behavior among this segment with PD. Table 10 synthesizes these results.

Eating behaviors among individuals with Parkinson’s disease are notably altered, often leading to compulsive eating and food cravings. These changes are influenced by the disease’s progression and the effects of dopamine replacement therapy. Eating behaviors in Parkinson’s disease include episodes of compulsive eating, food addiction, and binge eating disorder, with significant cravings and impulsivity observed in affected patients [79,80,81,82]. In the study by Chazeron et al. (2019), approximately 21.6% of PD patients experience episodes of out-of-control eating, while 39.2% meet the criteria for food addiction without binge eating disorder [79]. Furthermore, compulsive eating is frequently linked to dopamine replacement therapy, which can alter the hedonic value of food and lead to increased cravings for specific food types, particularly sweets [81,82]. Some patients often prefer sweet snacks (i.e., candies and ice cream), which can lead to undesired weight gain [82]. In the study by Zahodne et al. (2011), 8.3% of patients diagnosed with PD developed binge eating disorder [83]. Consumption of sugar-rich foods is most likely due to the dopaminergic dysregulation in the brain’s reward system, as the increased consumption of sugar-rich foods stimulates reward pathways in PD patients [80]. On the other hand, 90% of PD patients are affected by impairments in olfactory function, which can further alter food preferences and reduce the enjoyment of food, leading to decreased appetite and interest in eating [84]. A study by Isella et al. (2003) reported physical anhedonia, a deficit in the sensory experience of food, in 40% of PD patients [85]. Having said that, due to sensory deficits, PD patients have been reported to prefer foods with stronger flavors, such as sweet foods, or carbohydrates like cakes, chocolate, and ice cream [86].

**Table 10:** Eating behavior among segments with Parkinson’s disease.

Authors/Title	Aim	Methodology/Participants	Main Findings Regarding Anhedonia
Chazeron, Durif, Chéreau-Boudet, Fantini, Marques, Derost, Debilly, Brousse, Boirie, & Llorca, 2019 [79]. “Compulsive eating behaviors in Parkinson’s disease”.	The aim was to study the eating disorders among PD patients.	Clinical study/51 PD patients	Among the PD patients who experienced modified dietary habits following diagnosis, few exhibited full binge eating disorders (BED) criteria (3.9%). However, 21.6% of patients experienced episodes of out-of-control eating with a large quantity of food in a short time and 39.2% satisfied the food addiction (FA) criteria without binge eating disorder. Food cravings more than once a week were experienced in approximately half of the population including all FA patients.
Aviles-Olmos et al., 2013 [80]. “Exenatide and the treatment of patients with Parkinson’s disease”.	The aim is to investigate the potential therapeutic effects of exenatide, a GLP-1 receptor agonist, on symptoms or progression of Parkinson’s disease.	Clinical study/45 subjects	Many participants experienced a reduction in appetite, leading to weight loss over the course of the study. The GLP-1 receptor agonist mechanism of exenatide can alter reward-related eating behavior, potentially decreasing compulsive eating or cravings.
Fernagut, 2019 [81]. “Dopamine and eating behavior disorders in Parkinson’s disease: A complex recipe”.	The study aims to investigate the complex relationship between dopamine regulation and eating behavior disorders in individuals with Parkinson’s disease (PD).	Meta-analysis	Compulsive eating is frequently linked to dopamine replacement therapy, which can alter the hedonic value of food and lead to increased cravings for specific food types, particularly sweets.
Miwa & Kondo, 2008 [82]. “Alteration of eating behaviors in patients with Parkinson’s disease: Possibly overlooked?”	The aim was to assess food cravings and/or compulsive eating in PD patients, and the potential use of the dopamine replacement therapy.	Clinical study/60 patients	Among them, five (8.3%) patients exhibited characteristic alterations of food preference following the start of dopamine replacement therapy. One patient showed an undesirable weight gain. Of the five patients exhibiting food preference alterations, all showed increased preference to consume sweet snacks, although this alteration was not always associated with hyperphagia (eating too much).
Zahodne et al. 2011 [83]. “Binge eating in Parkinson’s disease: prevalence, correlates and the contribution of deep brain stimulation”.	The purpose of the present prospective study was to determine the prevalence of and factors associated with BED and subthreshold binge eating in Parkinson’s disease.	Clinical study/96 PD patients	Center, one (1%) met diagnostic criteria for binge-eating disorder. Eight (8.3%) exhibited subthreshold binge eating. More overeaters met psychometric criteria for at least one additional impulse-control disorder (67% vs. 29%). No more overeaters than non-overeaters were taking a dopamine agonist (44% vs. 41%).
Doty, 2012 [84]. “Olfactory dysfunction in Parkinson disease”.	The aim is to investigate the role and characteristics of olfactory (smell) dysfunction in individuals with Parkinson’s disease (PD).	Clinical study/healthy subjects (control group), and PD patients	Due to olfactory dysfunction, individuals with Parkinson’s may experience a diminished sense of smell, which can reduce the pleasure derived from eating, often leading to a lower appetite and reduced food intake. Many individuals with PD show a preference for foods with stronger flavors, as olfactory impairment might diminish their ability to detect subtle tastes and aromas.
Isella, Iurlaro, & Piolti, 2003 [85]. “Physical anhedonia in Parkinson’s disease”.	The aim of this study was to formally assess the prevalence and correlates of physical anhedonia in PD patients compared with normal controls.	Clinical study/25 PD patients and 25 control subjects	Anhedonia levels were significantly higher in PD patients with respect to controls, although not extremely elevated; prevalence rate was 40% for parkinsonians, while no anhedonics were found among controls.
Meyers, Amick, & Friedman, 2010 [86]. “Ice cream preference in Parkinson’s disease”.	The study aims to investigate how individuals with Parkinson’s disease may have specific preferences or changes in their taste and food preferences, particularly regarding ice cream.	Meta-analysis	Due to sensory deficits, PD patients have been reported to prefer foods with stronger flavors, such as sweet foods, or carbohydrates like cakes, chocolate, and ice cream.

## 4. Discussion

### 4.1. Main Findings Regarding Anhedonia Amongst Different Segments

The main aim of the present narrative review was to investigate the prevalence of anhedonia among various segments and understand the underlying factors contributing to that. Key results from the present study confirmed that anhedonia is a core symptom of several psychiatric illnesses, particularly schizophrenia and bipolar disorder. It is a prominent symptom of their clinical diagnosis [38], with studies indicating the presence of anhedonia in approximately 40–60% of patients with schizophrenia, with some reports suggesting even higher prevalence in specific clinical populations [39]. Additionally, approximately 37% to 80% of patients with mood disorders report anhedonic symptoms [38,40]. Anhedonia is also prevalent among patients with type 2 diabetes, with up to 30% of the patients reporting it as a common symptom [56,57]. Evidence from a systematic review by Harrison et al. (2010) suggests that anhedonia is a common feature of eating disorders and may be present in more than 50% of affected individuals [67]. Lastly, Parkinson’s disease patients consistently reported anhedonia as a primary symptom, with prevalence rates between 16.5 and 70% [74]. This was found primarily through more patients reporting a lack of motivation, social engagement, and lower quality of life, resulting in anhedonia being found to be a primary symptom of various conditions. Also, research indicates that anhedonia not only contributes to the severity of depression but also operates independently, necessitating targeted treatment approaches [38,39,40].

Several studies have demonstrated the prevalence of anhedonia and depression as a significant concern in various health conditions [87]. In the study by Wardani and Lesmana (2024), anhedonia is defined as the loss of interest or pleasure in activities once found enjoyable, and it is a primary diagnostic criterion for MDD [88]. Moreover, anhedonia was found in 45% of all patients, yet in the depressed sample, anhedonia was reported by 36.9% of subjects [38].

Further, research highlights dysfunctions in the neural networks responsible for processing rewards, mainly linked to altered activity in key brain regions involved in reward processing, particularly the mesocorticolimbic dopamine system. These impairments are especially pronounced in individuals with major depressive disorder (MDD) and schizophrenia, where anhedonia often extends beyond a mere absence of pleasure to more complex disruptions in reward processing, such as anticipation, consumption, and learning from rewards [2,3]. In the study by Liang et al. (2022), the authors highlight that anhedonia is characterized by impairments in anticipatory pleasure and integration of reward-related information in MDD. In contrast, anhedonia in schizophrenia is associated with neurocognitive deficits in representing the value of rewards [89]. Research also suggests that among patients with mood disorders (20–25% of patients), anhedonia is caused by dysregulation in reward processing systems, which are implicated in both mood regulation and motivation [14,41]. The same conclusions have been found in diabetic and MDD patients, where common symptoms (dysregulation of the hypothalamic–pituitary–adrenal (HPA) axis, chronic inflammation, and insulin resistance) are affected by dopaminergic pathways related to the brain’s reward system, thus contributing to anhedonia [60,61]. Neurobiological studies have suggested that alterations in dopamine pathways, critical for reward processing, may underlie the co-occurrence of anhedonia and eating disorders, particularly in AN and BN [70]. Der-Avakian and Markou (2012) explored dopamine’s role in transferring positive incentive value from rewards to cues that predict them among patients with Parkinson’s disease [4]. Their research suggests that disturbances in dopaminergic transmission and the mesolimbic circuit, particularly the progressive degeneration of dopaminergic projections, may contribute to anhedonic responses (lack of motivation and appetite), especially in patients with Parkinson’s disease [4].

It should be noted that the evidence regarding the prevalence and clinical significance of anhedonia across the reviewed populations is supported by a combination of systematic reviews, meta-analyses, and individual observational studies. Overall, the presence of anhedonia in conditions such as schizophrenia, mood disorders, eating disorders, and Parkinson’s disease is supported by relatively consistent evidence across multiple study designs [38,39,68,75].

Having said that, anhedonia is now recognized as a core feature of reward deficits in various segments with anhedonic traits. It involves the inability to experience pleasure, diminishing motivation, altered reward expectations, depressive syndrome, and making decisions based on past rewarding experiences [4,19].

### 4.2. Main Findings Regarding Eating Behavior and Food Choices

A secondary aim of this study was to investigate eating behaviors among various segments with anhedonic traits. Although eating behavior varies among segments with anhedonic traits, some similarities exist. Overall, there is a tendency towards irregular meal patterns, food cravings, and increased snacking, which can increase the prevalence of disordered eating behaviors [73]. In the study by Sankaranarayanan et al. (2021), patients with schizophrenia frequently presented disordered eating behaviors (DEBs), particularly binge eating (4.4% to 45%), food cravings (16.1% to 64%), food addiction (27% to 60.6%), and night eating (4% to 30%). These problematic eating behaviors can contribute to nutritional deficiencies and/or excessive weight gain, significantly impacting their health and quality of life [42]. These findings may suggest that anhedonia and other negative symptoms contribute to altered eating behavior by reducing the anticipated pleasure associated with food while simultaneously increasing vulnerability to maladaptive reward-seeking behaviors [2,3,42]. Another study further found that the prevalence of DEBs was noted among 41.5% of schizophrenia patients, which was significantly higher than the 10.3% in control groups [43]. Additionally, DEBs in schizophrenia patients range from 4.4% to 45% for binge eating and 16.1% to 64% for food cravings [42]. On the other hand, DEBs in bipolar patients are significantly higher during manic and depressive episodes [48]. There is also a tendency for impulsive eating, which can lead to binge-like behaviors or erratic eating schedules [48]. Moreover, many patients engage in emotional eating, with food being used as a coping mechanism to deal with negative emotions [50] and a way to improve their mood temporarily [48]. These segments are characterized by a high consumption of processed foods, sugars, and fats and a lower intake of fruits, vegetables, and fiber [44]. Similar eating behaviors can be seen across the segments with MDD; a study found that 19% to 44% of participants reported binge eating episodes [51]. Moreover, a study by Kunugi (2023) found that patients with a major depressive disorder often exhibit unhealthy eating behaviors, including energy overload, skipping breakfast, following a Western diet, and high consumption of ultra-processed foods [87]. Among this segment, disruptions in the reward system are believed to affect food intake by increasing impulsivity and a higher intake of unhealthy options like lard and sugar [48,55]. Additionally, MDD patients, especially during depressive episodes, will consume significantly less healthy foods and essential nutrients, such as fruits, vegetables, and fish [55]. In mood disorders, anhedonia may contribute to eating disturbances through alterations in reward sensitivity and motivation; however, the resulting behaviors may vary considerably, ranging from overeating and food cravings to reduced appetite and poorer diet quality [48,51,55]. Eating behaviors among individuals suffering from diabetes mainly involve emotional, external, and restrained eating patterns. Generally, our analysis found that females showed higher emotional and restrained eating tendencies than men, influencing their diet quality and health outcomes [63]. Findings from an observational study by Arhire et al. (2024) suggest that emotional and external eating behaviors are prevalent among diabetic patients, leading to higher calorie and lipid intake. In contrast, restrained eating showed no correlation with a specific type of dietary choice [62]. Additionally, females and males reported binge eating and night eating, resulting in longer diabetes duration and complicating glycemic control and weight normalization [65].

Moreover, 44.6% of diabetic patients exhibited poor eating behaviors, characterized by excessive intake of carbohydrates, which negatively impacted blood glucose levels [90]. These findings suggest that anhedonia may interact with metabolic and psychological factors in diabetes, potentially influencing emotional eating, food choices, and adherence to dietary recommendations [60,62,63]. Among patients with EDs, the disruptions in the brain’s reward processing systems cause the inability to derive pleasure from intrinsic rewards, such as eating or social interactions. They may further reinforce the disordered behaviors, such as food restriction, binge eating or purging, by diminishing the intrinsic reward value of food or other pleasurable activities [71]. This is especially seen among AN patients with severe caloric restrictions or extreme dieting practices [71]. Individuals suffering from bulimia nervosa disorder reported fewer meals and frequent overeating, while binge-eating disorder participants had a higher frequency of binge eating episodes [72]. Among individuals with eating disorders, anhedonia may manifest differently depending on the disorder subtype, contributing either to restrictive eating patterns through diminished food-related pleasure or to binge-eating behaviors through altered reward valuation and food-related motivation [70,71,72]. When it comes to eating behaviors among patients with Parkinson’s disease, they report episodes of compulsive eating, food addiction, and binge eating disorder, with significant cravings and impulsive consumption. An individual clinical study by Zahodne et al. (2011) reported that 8.3% of patients diagnosed with PD developed binge eating disorder [83], and in the study by Chazeron et al. (2019), approximately 21.6% of PD patients experienced episodes of out-of-control eating, while 39.2% met the criteria for food addiction without binge eating disorder [79]. Further, research highlighted that 90% of PD patients are affected by impairments in olfactory function, leading to decreased appetite and interest in eating [84] and a preference for foods with stronger flavors, such as sweet foods or carbohydrates like cakes, chocolate, and ice cream [86]. In Parkinson’s disease, alterations in reward processing, together with sensory impairments such as olfactory dysfunction, may contribute to changes in appetite, food preferences, and eating behavior [4,84,86].

In contrast, evidence regarding specific eating behavior characteristics and their relationship with anhedonia is more heterogeneous. While some findings are supported by systematic reviews and broader evidence syntheses, several observations regarding food cravings, emotional eating, binge eating, restrictive eating, and food preferences originate from individual observational or clinical studies and should therefore be interpreted with appropriate caution [42,62,79,87].

Overall, the findings suggest that anhedonia is associated with alterations in eating behavior across a wide range of psychiatric, neurological, and metabolic conditions; however, the manifestations differ considerably between populations [2,3,4,8]. While some individuals exhibit increased food cravings, emotional eating, binge eating, or compulsive food-seeking behaviors, others display reduced appetite, food restriction, or diminished pleasure derived from eating [42,51,62,71,79]. These findings indicate that anhedonia is unlikely to influence eating behavior through a single mechanism. Rather, disruptions in reward processing may interact with condition-specific psychological, physiological, and environmental factors to produce distinct eating-related outcomes [2,3,89]. Therefore, although anhedonia appears to be a common feature across several conditions, its impact on eating behavior should be interpreted within the context of the underlying disorder.

## 5. Limitations

A limitation of this review is the heterogeneity of the included evidence, which comprises systematic reviews, meta-analyses, and observational studies across diverse clinical populations. As a result, the strength of evidence varies between topics. While findings from systematic reviews and meta-analyses generally provide stronger evidence, findings from individual observational studies should be interpreted with appropriate caution. Additionally, as this review was conducted using a narrative approach, a formal risk-of-bias assessment was not performed.

## 6. Conclusions

The present narrative review explored the prevalence of anhedonia across different clinical populations while at the same time exploring whether these segments possess certain eating behavioral characteristics. Additionally, this review provides a knowledge basis for further investigation of the relationship between anhedonia, reward processing, and eating behavior. Overall, the literature identified several psychiatric, neurological, and metabolic conditions in which anhedonia is prevalent and suggested potential links between anhedonic traits and alterations in eating behavior.

The findings from the narrative review underscore anhedonia as a prominent and complex symptom within psychiatric and neurological conditions such as schizophrenia, bipolar disorder, Major Depressive Disorder, diabetes, and Parkinson’s disease. Across these populations, anhedonia has been associated with impairments in reward-related processes, including anticipatory and consummatory aspects of reward. Although several studies have proposed the involvement of neural reward pathways, particularly dopaminergic systems, the extent to which these mechanisms contribute to anhedonia may vary across conditions and may differ between clinical populations.

Moreover, this review also suggests that anhedonia may be associated with a range of disordered eating behaviors, including binge eating, emotional eating, food cravings, restrictive eating, and reduced pleasure derived from food. However, the nature of these associations differs considerably between populations, indicating that anhedonia is unlikely to influence eating behavior through a single mechanism. Rather, these relationships may involve interactions between reward-related processes and condition-specific psychological, physiological, and environmental factors.

Due to the current and future public health challenges, particularly concerning obesity, type 2 diabetes, eating disorders, and mental illness, it is crucial to broaden the understanding of how anhedonia and reward-related processes influence eating behavior. This is relevant to a fundamental knowledge-generating perspective but can possibly also be applied in contexts of dietary guidance for the diagnosed and undiagnosed populations with anhedonic traits and to support their well-being. Having said that, future research should integrate neurobiological, psychological, and behavioral assessments to more comprehensively investigate the multidimensional nature of anhedonia and its impact on eating behaviors across different clinical populations.

## Data Availability

The datasets generated for this study are available upon request to the corresponding author.
